# Evaluation of the Relation Between Hepatic Fibrosis and Basic Laboratory Parameters in Patients With Chronic Hepatitis B Fibrosis and Basic Laboratory Parameters

**DOI:** 10.5812/hepatmon.16975

**Published:** 2014-04-01

**Authors:** Nazlim Aktug Demir, Servet Kolgelier, Serap Ozcimen, Gokhan Gungor, Sua Sumer, Lutfi Saltuk Demir, Ahmet Cagkan Inkaya, Onur Ural

**Affiliations:** 1 Infectious Diseases and Clinical Microbiology Department, Selcuk University, Konya, Turkey; 2 Infectious Diseases and Clinical Microbiology Department, Adiyaman University, Adiyaman, Turkey; 3 Infectious Diseases and Clinical Microbiology Department, Konya State Hospital, Konya, Turkey; 4 Gastroenterology Department, Konya Education and Training Hospital, Konya, Turkey; 5 Public Health Department, Necmettin Erbakan University, Konya, Turkey; 6 Infectious Diseases and Clinical Microbiology Department, Hacettepe University, Ankara, Turkey

**Keywords:** Hepatitis B, Chronic, Biological Markers, Liver Cirrhosis

## Abstract

**Background:**

The hepatitis B virus is an important healthcare problem. According to current clinical practice, a liver biopsy is required for the diagnosis and treatment of chronic liver disease. However, a liver biopsy is an invasive, inconvenient procedure, which requires an expert pathologist opinion. Therefore requirement of biochemical tests, which are considered to indicate hepatic fibrosis and may be repeated easily, increases gradually today.

**Objectives:**

This study evaluated the correlation between hepatic fibrosis and routine laboratory values in patients with chronic hepatitis B.

**Patients and Methods:**

The files of 456 patients with CHB (chronic hepatitis B) who were referred to the infectious diseases and clinical microbiology clinic between January 2009 and March 2012 were screened retrospectively. Liver biopsy samples were examined according to Ishak scoring. Laboratory parameters and histopathology reports were recorded, and correlations between the fibrosis grade and laboratory parameters were analyzed.

**Results:**

There were 320 male and 136 female patients, with a mean age 36.7 ± 12.1 years. According to liver biopsy results, a low fibrosis score (stage 0-2) was detected in 281 patients (61.6%), and a high fibrosis score (stage 3-5) was detected in 175 patients (38.4%). Patients with a high fibrosis score had significantly higher ALT (alanine amino transferase), AST (aspartate aminotransferase), and HBV-DNA values and a significantly lower platelet count compared with those with a low fibrosis score (P = 0.001, 0.001, 0.025, and 0.001, respectively). A positive correlation was detected between the fibrosis score and age, BMI, HAI, ALT, and AST values, and a negative correlation was detected between the fibrosis score and albumin and platelet counts. In the regression analysis performed to evaluate the factors associated with high-stage fibrosis, fibrosis was determined to be associated with thrombosis, ALT, and gender. The results of the regression analysis demonstrated that the risk of fibrosis was 4.6 fold higher in men.

**Conclusions:**

According to the results obtained in our study, advanced age, higher BMI, AST, ALT, and HBV-DNA levels, and low albumin and platelet levels are correlated with advanced fibrosis in patients with CHB.

## 1. Background

The hepatitis B virus (HBV) is the most important cause of chronic hepatitis, cirrhosis, and hepatocellular carcinoma, and it is an important healthcare problem. There are over 350 million carriers of chronic HBV today, and about 500000 to 1200000 million people die due to complications caused by chronic hepatitis B (CHB) (1). Chronic liver disease is detected during the asymptomatic period with serological methods, which are used to analyze the patient’s immune status and virus level. If the disease is diagnosed during the chronic hepatitis period before the development of cirrhosis, treatment is possible with drugs that have been developed in recent years. The treatment response is more effective in cases with low-stage fibrosis. Therefore, together with biochemical and serological diagnostic methods that have been long been used, a histopathological assessment is important to detect necrosis, inflammation, and fibrosis, in particular, in liver tissue (2).

Percutaneous liver biopsy is the gold standard to show liver fibrosis today. However, the risk of complications with this invasive method is 1–5%, and the risk of mortality is 0.1 to 0.01% (3). Inconvenience application of liver biopsy on the patient in any place in any time by the physician, being and invasive procedure with complications are limiting factors for use. Drawbacks of a liver biopsy are the difficulty of needle biopsy on the liver, the presence of coagulation disorder, the heterogeneous distribution of histopathological findings in the tissue, and different interpretations of the findings (4). Due to these disadvantages, there is a need for noninvasive histological indicators in the assessment of patients with HBV. Research is ongoing on biochemical tests that may be an alternative to liver biopsy in clinical practice (5). Different studies have been carried out to provide noninvasive prediction of fibrosis. These include costly and nonroutine laboratory tests of, for example, bilirubin levels, prothrombin activity, aspartate aminotransferase (AST)/alanine amino transferase (ALT) ratios, apolipoprotein A1 levels, alpha-2 macroglobulin levels, and haptoglobulin levels. These methods are not convenient use by physicians, especially in resource poor settings.

## 2. Objectives

This study evaluated the correlation between hepatic fibrosis and routine laboratory values in patients with CHB.

## 3. Patients and Methods

### 3.1. Patients

The files of 456 patients with CHB who were referred to the Infectious Diseases and Clinical Microbiology Clinic between January 2009 and March 2012 were screened retrospectively. Patients who had not received any treatment for CHB before, were 18 years and older, and without co-infection and a history of alcohol use were included in the study.

### 3.2. Preparation for Liver Biopsy

The following hepatitis markers were investigated in all the patients prior to the biopsy: ALT, AST, HBV-DNA levels, platelet counts, and prothrombin time (PT) levels. Liver ultrasonography was performed. Patients with PT ≥ 1.5 INR and a platelet count ≤ 50.000/mm^3^ were excluded from the study.

### 3.3. Liver Biopsy

The biopsy criteria of the American Association for the Study of Liver Diseases (AASLD) were used. Liver biopsies that were sent to the pathology laboratory within a formaldehyde fixation solution with a prediagnosis of CHB were examined. All the samples were needle biopsy material, and their lengths were 1-3 cm. Sections from the samples, which were stained by hematoxylin-eosin, reticulin, and Masson trichrome, were examined under light microscopy by two pathologists using the Ishak modified histological activity index (HAI) (6). Fibrosis score was recognized as follows: F 0-2: low- stage fibrosis, F 3-5: high- stage fibrosis.

### 3.4. Microbiological and Biochemical Parameters

Biochemical parameters were analyzed with Abbott-branded commercial kits (Ci 16000 Abbott, Germany). Hematological parameters were analyzed with CELL-DYN 3700 (USA). The prothrombin time (PT) was tested by the clotting method (STA Compact, France). Levels of HBsAg, anti-HBs, HBeAg, and anti-HBe were studied with macro ELISA (Abbott AXSYM, SYSTEM, Germany). HBV-DNA test results analyzed by real time reverse transcriptase PCR (ICycler IQ Real-time PCR; BioRad, USA) were screened retrospectively.

### 3.5. Ethics

This work was carried out in accordance with the Declaration of Helsinki (2000) of the World Medical Association. Approval was obtained from the ethical committee of Adiyaman University (2012/02-4.3). Informed consent was received from all the patients involved in this study.

### 3.6. Statistical Analyses

Data on the study participants were analyzed using SPSS 18.0 software. For descriptive statistics, the mean ± standard deviation was used. Categorical data were analyzed using a Chi-square test, and continuous data were assessed with a t-test in the independent groups. Pearson’s correlation analysis was used to establish the correlation of the biochemical parameters with the fibrosis. Forward logistic regression analysis was performed to determine the factors associated with fibrosis. A value of P < 0.05 was considered statistically significant.

## 4. Results

There were 320 male (70.1%) and 136 female (29.9%) patients, with a mean age of 36.7 ± 12.1 years. According to the liver biopsy results, a low fibrosis score (stage 0-2) was detected in 281 patients (61.6%), and a high fibrosis score (stage 3, 4, or 5) was detected in 175 patients (38.4%). The fibrosis score was higher in male than in female patients (P = 0.010). The distribution of the patients according to fibrosis stage is given in Table 1. HBeAg was positive in 223 patients, anti-HBe was positive in 229 patients, and both HBeAg and anti-HBe were positive in 4 patients. Higher HBeAg positivity was detected in patients with low-stage fibrosis than in those with high- stage fibrosis (P = 0.001), and anti-HBe positivity was higher in patients with high- stage fibrosis than in those with low- stage fibrosis (P = 0.001). Age, AST, ALT, and HBV-DNA were higher and the platelet count was lower in patients with high- stage fibrosis than in patients with low- stage fibrosis. The demographic and biochemical features of the patients are shown in Table 2. There was a positive correlation between fibrosis score and age, body mass index (BMI), AST, ALT, HBV-DNA, and HAI. However, there was a negative correlation between the fibrosis score and albumin and platelet counts (Table 3). In the regression analysis performed to evaluate the factors associated with high-stage fibrosis, fibrosis was determined to be associated with thrombosis, ALT, and gender. The results of the regression analysis demonstrated that the risk of fibrosis was 4.6 fold higher in men (Table 4).

**Table 1.** Distribution of Patients According to Stage ^a^

Stage	Patients
**Stage 1**	29 (6.3)
**Stage 2**	252 (55.2)
**Stage 3**	144 (31.5)
**Stage 4**	18 (3.98)
**Stage 5**	13 (3.02)

^a^ Data are presented in No. (%).

**Table 2.** Demographic and Biochemical Features of Patients ^a^

Parameters	Low Fibrosis Group (n = 281)	High Fibrosis Group (n = 175)	P Value
**Age, y**	34.1 ± 11.5	40.9 ± 12.0	0.001
**Platelet, × 10^3^/mL**	277933 ± 158850	219054 ± 94215	0.001
**ALT, IU/L**	99.1 ± 103.1	139.7 ± 79.2	0.001
**AST, IU/L**	57.2 ± 32.4	72.7 ± 34.4	0.001
**AFP, IU/mL**	3.3 ± 1.8	3.5 ± 2.3	0.475
**Albumine, g/dL**	3.5 ± 0.3	3.5 ± 0.5	0.700
**ALP, IU/L**	86.8 ± 14.8	87.3 ± 13.1	0.746
**GGT, IU/L**	31.3± 11.5	30.8 ± 11.4	0.639
**HBV DNA, copy/mL**	9.4 × 10^8^ ± 3.3 × 10^9^	3.6 × 10^8^ ± 1 × 10^10^	0.025

^a^ Abbreviations: AFP, Alpha feto-protein; ALP, Alkaline phosphatase; ALT, alanine amino transferase; AST, alanine amino transferase; GGT, Gama glutamyltransferase.

**Table 3.** Correlations With Fibrosis Score^a^

	Pearson Correlation Analysis	
	r	P Value
**Age, y**	0.185	0.001
**Platelet**	-0.233	0.001
**BMI**	0.155	0.001
**ALT, IU/L**	0.210	0.001
**AST, IU/L**	0.267	0.001
**Albumin, g/dL**	-0.128	0.006
**ALP, IU/L**	0.008	0.857
**GGT, IU/L**	-0.055	0.239
**HBV DNA, copy/mL**	0.087	0.064

^a^ Abbreviation: BMI, body mass index.

**Table 4.** Factors Associated With Severe Fibrosis ^a^

	B	SE	Wald	P Value	Exp(B)	%95 CI	
						Lower	Upper
**Gender **	1.53	0.77	3.88	0.49	4.62	1.00	21.22
**Platelet**	0.01	0.01	24.72	0.001	1.00	1.00	1.00
**ALT**	0.11	0.006	3.75	0.50	1.01	1.00	1.02

^a^ Abbreviations: B, The coefficient for the constant; SE: Standart error; Exp (B), Exponentiated logistic coefficients.

## 5. Discussion

Several studies have been conducted on noninvasive tests that may an alternative to liver biopsy (7-13). In these studies, researchers tried to find methods that show fibrosis in the most accurate way by using basic biochemical examinations. However, these tests or methods that may indicate liver fibrosis in patients with chronic hepatitis have been studied in patients with chronic hepatitis C and hepatic steatosis (7, 9, 14-16). Studies of CHB are very limited. In our study, some of the basic tests that may be associated with fibrosis were compared to patients’ fibrosis stage in 456 patients with CHB diagnosis.

As for the studies conducted on this subject, in a study including 276 cases with CHB, Mohamadnejad et al. (17) found that the average age was lower in patients with mild fibrosis than in those with severe fibrosis. The platelet count and the albumin level were lower in those with severe fibrosis, and HBV DNA and ALP levels were significantly higher. In HBeAg-negative cases, in contrast to expectations, the serum ALT level was not higher in cases with severe necroinflammation and fibrosis, suggesting that it is not a significant predictor of fibrosis. The same study found that thrombocytopenia was significantly associated with ALT elevation and severe necroinflammation in HBeAg-positive patients (17). Similar to these data we also found in our study that age, AST, ALT, and HBV-DNA were higher and the platelet count was lower in patients with high-stage fibrosis than in patients with low- stage fibrosis. In addition, there was a positive correlation between fibrosis score and age, body mass index (BMI), AST, ALT, HBV-DNA, and HAI. However, a negative correlation was detected between the fibrosis score and albumin and platelet counts.

A significant reduction is observed in platelet count especially in the development of cirrhosis in the advanced stages of chronic hepatitis. Consistent with this observation, there was a significant difference in the platelet level between the two groups in our study (P = 0.001). In parallel with our findings, in a study of 1143 patients with CHC, Iacobellis et al. (16) reported that a platelet threshold value of < 140.000/mm^3^ was a sensitive indicator of fibrosis. In addition, no correlation was found between the fibrosis score and the viral load in our study (P > 0.05). Similarly, in a study of 200 patients, Lu et al. (18) detected no correlation between the viral load, inflammatory activity, and the fibrosis score. In this study, according to the regression analysis, thrombosis, ALT and gender were found to be factors associated with severe fibrosis. Risk of fibrosis was determined to be 4.6 fold higher in men. Our literature review has not revealed any study on this subject. This result was associated with the proportion of men (70.1%) in the patient population. Still, further studies are needed to clarify this point.

The most important limitation of our study is that a limited number of laboratory parameters were evaluated to detect fibrosis due to the retrospective nature of our study. On the other hand, the high number of cases and inclusion of patients with CHB are important features of our study. Consequently, requirement of biochemical tests which are considered to indicate hepatic fibrosis and may be repeated easily increases gradually today. Retrospective studies where liver biopsies and biochemical findings are compared are instructive to reveal new tests. According to our study, advanced age, a higher BMI, higher AST and ALT levels, and low albumin and platelet levels are correlated with advanced fibrosis in patients with CHB Therefore; these parameters may be assessed as indicators of advanced stage fibrosis in patients with CHB.
